# National associations between pandemic electronic benefit transfer (P-EBT) implementation practices and state characteristics

**DOI:** 10.3389/fpubh.2026.1886776

**Published:** 2026-08-10

**Authors:** Fernando R. Mena-Carrasco, Erika Estrada-Ibarra, Wura Olawole, Rachel Cahill, Emily Calman, Tiffany Hoang, Lucine Francis, Elizabeth A. Stuart, Laura J. Samuel

**Affiliations:** 1 School of Nursing, Johns Hopkins University, Baltimore, MD, United States; 2 Department of Biostatistics, Mailman School of Public Health, Columbia University, New York, NY, United States; 3 Rachel Cahill Consulting, Cleveland, OH, United States; 4 Johns Hopkins Center for School Health, Baltimore, MD, United States; 5 Johns Hopkins University Bloomberg School of Public Health, Baltimore, MD, United States

**Keywords:** child health, food insecurity, pandemic, P-EBT, policy implementation, school meals, summer food assistance, SUN Bucks

## Abstract

**Background:**

The Pandemic Electronic Benefit Transfer (P-EBT) program replaced free and reduced-price school meals for low-income children during school closures and summers from 2020 to 2023. Evidence indicates that P-EBT disbursements were associated with a 40% reduction in food hardship and better maternal mental health among low-income households with children. As states had discretion in implementing the program, approaches varied across states. This study characterized patterns of P-EBT implementation and disbursement across all 50 states and Washington, D.C., in relation to state characteristics.

**Methods:**

We examined three outcomes, namely timing of first P-EBT disbursement in 2020, inconsistency of summer P-EBT participation through 2023, and annual per capita P-EBT disbursement amounts from 2020 to 2023, using ordered logistic, logistic, and linear regression models. Independent variables included measures of state economic resources, sociopolitical characteristics, and indicators of need for food assistance. To account for regional cost-of-living differences, we conducted a sensitivity analysis using a real state minimum wage adjusted for Regional Price Parity (RPP).

**Results:**

In unadjusted analyses, Republican gubernatorial affiliation was associated with later first disbursement, lower per capita disbursement, and inconsistent summer participation. In adjusted models, no state characteristics were significantly associated with the timing of the first disbursement. Each additional month of prior-year Supplemental Nutrition Assistance Program (SNAP) emergency allotments was associated with lower odds of inconsistent Summer P-EBT participation [odds ratio (OR) = 0.72, 95% confidence interval (CI): 0.57–0.90]. Higher Medicaid participation rates [regression coefficient (B) = $1,932.40, *p* < 0.005] and more months of SNAP emergency allotments [B = $23.70, *p* < 0.05] were associated with higher per capita disbursements. Sensitivity analyses using the RPP-adjusted minimum wage yielded materially unchanged results.

**Conclusion:**

P-EBT was implemented more comprehensively in states with Democratic governors and in states with more comprehensive SNAP emergency allotments, Medicaid, and other safety-net programs. Indicators of economic conditions, such as poverty and unemployment rates, were not associated with implementation patterns. Findings underscore the role of political context in shaping food assistance implementation, with direct implications for the SUN Bucks program, which uses practices similar to P-EBT but is not yet implemented by all states.

## Introduction

The Pandemic Electronic Benefit Transfer Program (P-EBT) was an important new food assistance program initiated in March 2020 to replace free and reduced-price meals for low-income, school-aged children who were not receiving meals due to school closures (1). Although the program ended in 2023, its administrative structure informed the design and implementation of the federal SUN Bucks program launched in 2024 (2). Examining state-level P-EBT implementation practices can provide insight into strengthening the SUN Bucks program and the rapid deployment of large-scale federal food assistance programs intended to rapidly ameliorate food insecurity among eligible households. P-EBT provided eligible children with the cash equivalent of missed school breakfasts and lunches (3, 4). All states and Washington, D.C., implemented P-EBT for school-aged children by the end of April 2020. The program was expanded in October 2020 for children under 6 years of age who attended childcare facilities that were closed or operating with reduced hours (5). By the summer of 2021, P-EBT benefits had been disbursed to more than 30 million children (6), ultimately totaling more than $70 billion in benefits issued by 2023 (7). Recent evidence has shown that the disbursement of P-EBT benefits reduced food hardship by 40% for low-income households with children and improved maternal mental health (3).

Reductions in food hardships are important to child and adolescent health promotion and protection. Children experiencing food insecurity tend to have poorer parental-rated health (8) and a higher risk of asthma, depression, and other health-related economic hardships (9–11). Food insecurity has also been associated with more behavioral problems, poorer school attendance among school-aged children (8, 12, 13), and worse mental health outcomes among adolescents (14). Therefore, addressing food insecurity through primary prevention initiatives, such as P-EBT, is a critical step in protecting child health and development while reducing the risk of adverse health outcomes across the life course (15, 16).

Although P-EBT was federally funded, states were granted discretion in implementing the program, introducing meaningful variability in key program characteristics (4, 17, 18). Under this federal flexibility, each state’s P-EBT plan was developed and submitted for approval by the U.S. Department of Agriculture (USDA) and state executive-branch agencies, typically the same offices administering the Supplemental Nutrition Assistance Program (SNAP), in coordination with the state education agencies. Decisions about related pandemic flexibilities were generally tied to the Governor’s maintenance of a state emergency declaration (19). As the program was new, states differed in how quickly they developed plans, integrated data from schools and food assistance programs for eligibility verification, and disbursed benefits (17, 18). These administrative differences meant that benefits were disbursed within days of program implementation in some states, while in other states, initial disbursements were delayed by months (3, 17). Studying state characteristics associated with the speed of initial disbursements is important because prior research has shown that households in states with faster payments experienced lower food insufficiency (3, 4). States also varied in whether they consistently participated in P-EBT during the summer months, when food hardship typically increases for low-income children (3, 20, 21). Given the variation across states in program uptake and speed of disbursement, there were differences in the amounts of benefits disbursed each year (2, 3). However, there is a gap in identifying the state-level characteristics, such as economic resources, sociopolitical factors, and the need for food assistance, that played a role in the timely and consistent implementation of a new national food assistance program. Such information is needed to inform the implementation of future new programs and to improve efficiencies for existing similar programs, such as the new SUN Bucks program (2, 22).

There is broad consensus in the policy research literature that implementation decisions are multifactorial, shaped by economic, social, and political conditions operating at both the state and federal levels (23–27). However, the extent to which specific state-level characteristics (e.g., economic resources, indicators of population need, and sociopolitical factors) are associated with differences in P-EBT implementation remains poorly understood. Accordingly, the purpose of this study was to characterize the patterns of P-EBT implementation across all 50 states and Washington, D.C., from 2020 through 2023 in relation to state characteristics.

## Study materials and methods

### Outcome variables

Information about P-EBT implementation was extracted by two trained research assistants (FM-C and EE-I) from publicly available memoranda approved by the USDA for each state and Washington, DC, between 2020 and 2023, as well as from disbursement data reports. Following the completion of the initial data entry, all data were reviewed independently by another set of two trained research assistants (JR and WO) to ensure accuracy, and no discrepancies were found. A food policy expert (RC) was included in the study team to provide insight into the broader food policy environment in which pandemic-related changes were implemented and to provide guidance on the practical implementation of pandemic-related changes across states. This guidance informed the selection of information for data extraction and the operationalization of measures.

State P-EBT implementation practices were captured using three variables, selected to capture the speed, continuity, and reach of implementation, respectively. First, using data obtained from the USDA memoranda for all 50 states and Washington, D.C., we generated a variable capturing the first calendar month of P-EBT disbursements for each state in 2020. Second, we generated a binary indicator that captured whether states inconsistently participated in summer P-EBT programs. Inconsistent summer participation was defined as states that did not participate in all summer P-EBT program issuances (2021 and 2022 for school-aged and preschool-aged children, respectively, and 2023 for school-aged children). Third, annual per capita P-EBT disbursement amounts were estimated in two steps. First, annual state-level disbursements were calculated by summing monthly P-EBT disbursement amounts across the calendar year (January to December) using USDA data. Second, because children in households with incomes <185% of the federal poverty threshold (FPT) were eligible for P-EBT in all states, per capita disbursement amounts were calculated by dividing annual state disbursements by the number of children living in households with incomes <185% of the FPT using U.S. Census population estimates for each year (28). This approach was developed for this study and grounded in federal eligibility criteria, as the 185% FPT threshold represents the national income cutoff for P-EBT eligibility, thus providing a standardized estimate of publicly available disbursement data relative to the population of children eligible for benefits in all states (29–31). Notably, not all income-eligible children used P-EBT benefits, and some children with incomes ≥185% of the FPT were eligible for P-EBT if they attended school in a low-income community that utilized the Community Eligibility Provision (32). Therefore, the actual per capita disbursement amounts may vary from the amounts estimated here; however, the current approach was used to standardize the P-EBT disbursement amounts among the subset of low-income children who were eligible for P-EBT anywhere in the US. Although additional implementation details were available in the USDA memoranda, they reflected intended rather than realized implementation practices and could not be verified based on available information. Therefore, we focused on three outcomes, each capturing a distinct and substantively important dimension of implementation, that we could corroborate through other sources.

### Independent variables

Independent variables were selected *a priori* because they may reflect the economic resources that support state capacity, sociopolitical characteristics that may influence implementation decisions, or the need for food assistance within states. Economic resource variables included per capita Gross State Product (GSP), the unemployment rate, and minimum wage. To account for regional cost-of-living differences, we constructed a real minimum wage variable for a sensitivity analysis by dividing the lagged nominal minimum wage variable by each state’s Regional Price Parity (RPP) index value, a spatial price index published by the U.S. Bureau of Economic Analysis (BEA) that reflects relative price differences across states (33–36). Per capita GSP values were collapsed into quartiles due to their skewed distribution and to facilitate interpretability across statistical models. Sociopolitical characteristics included whether the Governor was a Republican (yes/no), each state’s population size, and the cumulative number of months that SNAP emergency allotments were active in each state. SNAP emergency allotments were temporary federal supplements authorized in 2020 that raised participating households to the maximum monthly benefit for their household size. All states initially adopted them, but some states ended them earlier than the federal program, and this change was generally tied to the Governor’s maintenance of a state emergency declaration (19). As states with Republican Governors were more likely to opt out of SNAP emergency allotments, we treated the number of months of emergency allotments as a measure of the sociopolitical context (19). The poverty rate was included as an indicator of the need for food assistance. The Medicaid participation rate was included as a dual indicator, reflecting both underlying need for food assistance and state-level sociopolitical dynamics, given the partisan nature of Medicaid eligibility expansion across the country (37–39). Of note, the household food insecurity rate was not used as an independent variable because P-EBT benefits reduce food insecurity, and we were concerned about reverse causality (3). Data for the independent variables were obtained from the National Welfare Data compiled by the University of Kentucky Center for Poverty Research for 2019 through 2022 (40) and from the U.S. BEA for the 2019 RPP (34).

### Statistical analyses

State-level associations between P-EBT implementation outcomes and independent variables were first examined using bivariate analyses, including Spearman’s correlations, chi-squared tests, and bivariate linear regression models. Since the Governor’s political party affiliation may influence other state characteristics that depend on policy decisions, we examined associations between the Governor’s political affiliation and other state characteristics using two-sample *t*-tests prior to building multivariable models (Supplementary Table 1). Multivariable models were then adjusted for all independent variables listed above to test associations between economic resources and measures of need with P-EBT implementation outcomes. To ensure appropriate temporal ordering, outcomes were always modeled as a function of the prior-year independent variable values (t-1), such that, for example, 2020 outcomes were regressed on 2019 values for independent variables. As RPPs are spatial indexes with values constructed using 5-year averages of the Consumer Price Index, a single pre-pandemic and pre-program launch value (2019) was applied across all study years (35). The first month of P-EBT disbursement was analyzed using ordered logistic regression, where odds ratios under one indicate relatively earlier disbursements. Models for inconsistent Summer P-EBT participation in 2022 or 2023 were estimated using logistic regression with independent variable values from 2021 (t-1 to t-2) to preserve temporal ordering, as several states stopped participating in 2022. Annual per capita P-EBT disbursement amounts were analyzed using linear regression models, pooling data across the years 2020 through 2023. To account for repeated measures across years, the multivariable linear model for P-EBT disbursement amounts included indicators for year in addition to the independent variables described above, and standard errors were clustered at the state level to account for repeated observations within states over time. A secondary model tested whether associations varied across calendar years by adding interaction terms between year and lagged state characteristics to the multivariable model, with joint Wald tests evaluating whether associations between lagged state characteristics and P-EBT disbursement amounts differed across calendar years. Finally, multivariable analyses stratified by year aimed to understand associations within each year of the P-EBT program. As a sensitivity analysis, the primary multivariable models as well as year-stratified models were re-estimated using the real (RPP-adjusted) minimum wage in place of the nominal minimum wage, with results reported in Supplementary Table 2, 3, respectively. All analyses were conducted using *StataNow/BE* version 19.5.

## Study results

### Descriptive statistics and bivariate results

The median month of first P-EBT disbursements in 2020 was May; values ranged from March (month 3) to August (month 8) (Table 1). Eight states (16%) inconsistently participated in Summer P-EBT in either 2022 or 2023. The overall mean annual per capita P-EBT disbursement was $780. States with a Republican Governor had a later first disbursement in 2020 (median of June vs. May, *p* < 0.05) and lower annual per capita disbursement amounts (coefficient = − $283.45, SE = 84.22, *p* < 0.001) than states with Democratic Governors. All eight states that inconsistently participated in Summer P-EBT had Republican Governors (Table 1). Republican-led states also had significantly lower Medicaid participation rates, minimum wages, per capita GSP, fewer months of SNAP emergency allotments, and higher poverty rates than Democratic-led states across the study period (*p* < 0.05 for all, Supplementary Table 1). Furthermore, states with higher unemployment rates in 2019 had earlier P-EBT disbursement in 2020 (Spearman *ρ* = −0.30, *p* < 0.05; Table 1). No other state characteristics were significantly associated with the first month of P-EBT disbursement. States that inconsistently participated in Summer P-EBT had fewer months of SNAP emergency allotments in 2021 (6.25 months vs. 6.9 months, *p* < 0.05; Table 1). No other state characteristics were significantly associated with inconsistent Summer P-EBT participation (Table 1). States with larger populations (coefficient = 0.0000189, SE = 0.00000579, *p* < 0.005), higher unemployment rates (coefficient = 178.64, SE = 43.25, *p* < 0.001), and Medicaid participation rates (coefficient = 2,280, SE = 556.1, *p* < 0.001), as well as those with higher per capita GSP levels (highest quartile vs. lowest coefficient = 344.67, SE = 139.9, *p* < 0.05), higher minimum wages (coefficient = 65.6, SE = 12.30, *p* < 0.001), and more months of SNAP emergency allotments (coefficient = 43.5, SE = 9.92, *p* < 0.001) in the prior year, disbursed higher annual per capita P-EBT amounts (Table 1). Poverty rates, however, were not significantly associated with annual per capita P-EBT disbursement amounts.

**Table 1:** Descriptive statistics bivariate relationships between state-level characteristics and P-EBT program implementation outcomes.

Median (Range)	Month of first P-EBT disbursement in 2020 (*n* = 51)		Inconsistent Summer P-EBT Participation 2022-2023^a^ (*n* = 51)			Annual per Capita P-EBT Disbursements ($) 2020–2023^b^ (*n* = 204)	
		*p*-value	Inconsistently participating *n* = 8	Consistently participating *n* = 43	*p*-value	Bivariate coefficient (SE)	*p*-value
Variable							
Governor political party affiliation^c^		**<0.05**			**<0.05**		
Democratic	Median = May (range: March–August)		0	23		Ref.	--
Republican	Median = June (range: March–August)		8	20		−283.45 (84.22)	**<0.001**
All governors	Median = May (range: March–August)		--	--		--	--

	Spearman’s *ρ*	*p*-value	Mean (SD)	Mean (SD)			
Medicaid participation rate	- 0.23	0.101	21% (2.3%)	26% (1%)	0.067	2280.1 (556.1)	**<0.001**
Per capita GSP $ (quartiles)	- 0.08	0.546	2.37 (1.30)	2.48 (1.09)	0.494	--	--
Quartile 1 (ref.)	--	--	--	--	--	(ref.)	--
Quartile 2	--	--	--	--	--	173.73	0.152
Quartile 3	--	--	--	--	--	124.13	0.367
Quartile 4						344.67	**<0.05**
Unemployment rate	- 0.30	**<0.05**	4.27% (0.49%)	4.94% (0.19%)	0.172	178.64 (43.25)	**<0.001**
State minimum wage	- 0.14	0.321	8.48 (0.59)	9.68 (0.38)	0.197	65.6 (12.30)	**<0.001**
Poverty rate	0.10	0.466	11.43% (1.065%)	11.12% (0.493%)	0.801	0.58 (13.75)	0.967
Population size	- 0.22	0.116	5,512,137 (3,495,460)	6,696,150 (1,062,839)	0.681	0.0000189 (0.00000579)	**<0.005**
Months with SNAP emergency allotments^d^	--	--	6.25 (0.526)	6.9 (0.051)	**<0.05**	43.5 (9.92)	**<0.001**

All independent variables were lagged 1 year relative to the outcome. Bold type indicates estimates that reached statistical significance (*p* < 0.05).

a Inconsistent summer participation was defined as states that did not participate in all Summer P-EBT program issuances (2021 and 2022 for school-aged and preschool-aged children and 2023 for school-aged children).

b Annual per capita P-EBT disbursement values shown are unadjusted bivariate regression coefficients estimated using December observations for all U.S. states and Washington, D.C., from 2020 through 2023, with year indicator variables included and standard errors clustered at the state level.

c Washington, D.C., had a Democratic mayor and was classified as Democratic for these analyses. *p*-values were obtained from a chi-squared test.

d SNAP Emergency Allotments were first implemented in 2020. This variable was not measured in 2019.

### Multivariable results

In multivariable models, no state characteristics were significantly associated with the first month of P-EBT disbursement (Table 2). Each additional month of SNAP emergency allotments in 2021 was associated with 28% lower odds of inconsistent Summer P-EBT participation in 2022 or 2023 (OR = 0.72, 95% CI: 0.57, 0.90; Table 2). Although having a Republican Governor was excluded from the multivariable regression model because of perfect prediction of the outcome (Table 1), no other state characteristics were significantly associated with inconsistent Summer P-EBT participation (Table 2). In multivariable models, a one-percentage-point increase in the Medicaid participation rate was associated with an additional $1,932.40 increase in annual per capita P-EBT disbursements (SE = 659.1, *p* < 0.005), and each additional month with SNAP emergency allotments was associated with $23.70 more in per capita disbursements (SE = 10.1, *p* < 0.05). No other state characteristics were statistically significantly associated with disbursement levels (Table 2).

**Table 2:** Multivariable associations between state characteristics and P-EBT program implementation outcomes.

	Month of first P-EBT disbursement in 2020^a^ (*n* = 51)		Inconsistent summer P-EBT participation 2022–2023^b^ (*n* = 51)		Annual per-capita P-EBT disbursements ($) December 2020–2023 (*n* = 204)	
Variable (t–1)	Odds ratio (OR)	95% confidence interval	Odds ratio (OR)	95% confidence interval	Coefficient (SE)^c^	*p*-value for year × t-1 variable interactions^d^
Medicaid participation rate	0.0004	(<0.001, 138.09)	0.01	---^f^	1932.4 (659.1)**	0.98
Per capita GSP ($)						0.36
Quartile 1 (ref.)	---	---	---	---	---	---
Quartile 2	1.60	(0.35, 7.35)	2.44	(0.11, 54.54)	109.8 (85.2)	---
Quartile 3	3.04	(0.51, 18.12)	1.32	(0.01, 137.1)	132.8 (99.7)	---
Quartile 4 (highest)	1.46	(0.13, 16.78)	15.52	(0.05, 4916.9)	148.8 (102.6)	---
Unemployment rate	1.01	(0.68, 1.50)	0.95	(0.19, 4.65)	91.3 (52.4)	0.808
State minimum wage	1.02	(0.71, 1.46)	0.92	(0.54, 1.55)	17.3 (16.9)	**0.021**
Poverty rate	1.24	(0.94, 1.64)	1.24	(0.71, 2.18)	- 52.6 (26.9)	0.055
Population size	0.99	(0.99, 1.00)	1.00	(0.99, 1.00)	0.0000117 (0.000006)	**<0.0001**
Months with SNAP emergency allotments^e^	---	---	**0.72**	(0.57, 0.90)**	23.7 (10.1)*	0.210
Republican governor^b^	2.03	(0.56, 7.35)	---	---	36.1 (183.5)	0.617

All independent variables were lagged 1 year relative to the outcome. However, models for inconsistent summer participation used 2021 covariate values to ensure exposure preceded the first potential occurrence of inconsistent participation in 2022. Bold type indicates estimates that reached statistical significance (*p* < 0.05).

* *p* < 0.05, ** *p* < 0.005. ^a^Estimates were obtained from an ordered logistic regression model, and month of first disbursement was captured as a calendar month, ranging from March (3) to August (8). Therefore, an odds ratio of <1 indicates earlier payment timing and odds ratios of >1 indicate later payment timing. ^b^Estimates were obtained from a logistic regression model. Models for inconsistent Summer P-EBT participation were estimated using independent variable values from 2021, thus ensuring that these values preceded prior to the outcome assessment. Republican Governor variable was excluded due to perfect prediction of the outcome; only states with Republican Governors inconsistently participated in Summer P-EBT. ^c^Estimates for annual per capita disbursements were obtained from pooled linear regression models using December observations for all U.S. states and Washington, D.C., from 2020 through 2023. Year indicator variables were included to model time. Standard errors were clustered at the state level. ^d^Estimates for annual per capita disbursements were obtained from pooled linear regression models using December observations for all U.S. states and Washington, D.C., from 2020 through 2023. Year indicator variables were included to model time. Model included interactions between year and all covariates, and joint Wald tests assessed whether associations differed across years. Standard errors were clustered at the state level. ^e^SNAP Emergency Allotments were implemented in 2020, and all states implemented the first year of issuance. Therefore, this variable was not included in the first month’s disbursement model. ^f^Confidence interval unreliable due to near-complete separation (value: 4.07 × 10^−18^, 4.76 × 10^13^).

Model testing interactions between year and lagged state characteristics for per capita benefit amounts found statistically significant heterogeneity across years for state minimum wage (joint Wald test *p* < 0.05) and population size (joint Wald test *p* < 0.01). No statistically significant temporal heterogeneity was observed for Medicaid participation (*p* = 0.98), per capita GSP quartiles (*p* = 0.36), unemployment rate (*p* = 0.80), poverty rate (*p* = 0.05), months of SNAP emergency allotments (*p* = 0.21), or states with a Republican Governor (*p* = 0.61; Table 2). States with larger populations in 2019 had larger P-EBT disbursements in 2020 (coefficient = 0.00004, *p* < 0.0005), and states with higher minimum wages in 2020 (coefficient = 180.3, *p* < 0.005) and more months of SNAP emergency allotments (coefficient = 113.1, *p* < 0.05) had larger P-EBT benefits disbursed in 2021 (Table 3).

**Table 3:** Cross-sectional annual models for per-capita P-EBT disbursements ($), December 2020–2023 (*n* = 51/year).

Variable (t–1)	2020 Coef. (SE)	2021 Coef. (SE)	2022 Coef. (SE)	2023 Coef. (SE)
*R* ^2^	0.53***	0.61***	0.33	0.29
Medicaid participation rate	2358.38 (1543.1)	1472.9 (2233.6)	1850.0 (888.3)*	2167.9 (869.5)*
Per capita GSP ($)				
Quartile 1 (ref.)	---	---	---	---
Quartile 2	182.52 (182.53)	140.4 (251.6)	67.5 (135.1)	229.32 (185.9)
Quartile 3	- 172.7 (206.5)	310.9 (317.4)	217.3 (151.4)	305.7 (186.1)
Quartile 4 (highest)	471.1 (290.2)	- 45.8 (302.8)	267.1 (162.9)	381.9 (195.7)
Unemployment rate	50.91 (43.6)	- 30.5 (114.1)	- 23.5 (76.8)	52.6 (69.7)
State minimum wage	- 33.14 (44.3)	180.3 (49.8)**	- 2.8 (19.3)	- 2.9 (18.1)
Poverty rate	- 60.62 (33.3)	36.9 (42.7)	20.7 (20.2)	- 29.2 (20.5)
Population size	0.00004 (0.00000923)***	0.0000118 (0.0000116)	−0.000000451 (0.00000548)	−0.00000384 (0.00000572)
Months with SNAP emergency allotments^a^	---	113.1 (50.4)*	16.1 (10.2)	25.8 (48)
Republican governor^b^	- 34.9 (155)	- 176.4 (191.6)	119.28 (98.7)	191.4 (106.9)

All independent variables were lagged 1 year relative to the outcome. Bold type indicates estimates that reached statistical significance (*p* < 0.05).

* *p* < 0.05, ** *p* < 0.005 *** *p* < 0.0005. ^a^SNAP Emergency Allotments were implemented in 2020, and all states implemented the first year of issuance. Therefore, this variable was not included in the 2020 model. ^b^Linear regression models were estimated separately for each year using December state-level observations (*n* = 51 per year). Standard errors were clustered at the state level.

Inferences were unchanged in sensitivity analyses that replaced the nominal minimum wage with the real (RPP-adjusted) minimum wage, except that population size was positively associated with per capita P-EBT disbursement amounts (coefficient = 0.0000398, SE = 0.000011, p < 0.005), and a poverty rate-by-year interaction reached significance in the pooled model (joint Wald test p < 0.05) (Supplementary Table 2). The poverty rate remained non-significant in every year-specific model across both specifications (Supplementary Table 2, 3).

## Discussion

This national study examined the relationship between state-level characteristics and differences in P-EBT implementation across all 50 states and Washington, DC, over the duration of the program. Republican-led states had later initial disbursements, inconsistent participation in Summer P-EBT, and lower annual disbursement amounts. Greater state participation in SNAP emergency allotments was also associated with more consistent participation in summer P-EBT and higher P-EBT disbursement amounts, suggesting that state-level food assistance program decisions are correlated. Notably, state characteristics that capture the need for food assistance, such as poverty rates, and those indicative of economic resources, such as unemployment rates, were not necessarily associated with P-EBT implementation, suggesting that programmatic decisions are not typically based entirely on program need. These findings contribute to the literature by identifying state characteristics that are associated with differences in P-EBT implementation, building on prior evidence that these program implementation decisions influenced food insecurity for low-income households with children (3).

Although this study found no association between having a Republican Governor and the study outcomes in multivariable models, the bivariate results may provide the least biased estimates of these relationships. Many of the state characteristics we controlled for in our models (e.g., minimum wage amounts, SNAP emergency allotment implementation, and Medicaid participation rates) are plausibly influenced by the Governor’s political affiliation because the

Governor’s offices are charged with leading the executive branch in each state and, therefore, with decision-making around programs and issues. In our sample, Republican-led states differed from Democratic-led states in multiple ways, consistent with prior evidence that these policy decisions cluster along partisan lines. Therefore, statistical adjustment for multiple state policy decisions may have biased associations between the Governor’s affiliation and P-EBT implementation decisions toward the null. Previous observational research on pandemic-era policy responses has documented similar patterns, a finding that Republican-led states tended to implement fewer COVID-19 public health policies, had lower COVID-19 testing rates, and experienced higher COVID-19 mortality rates in later pandemic phases, with these associations attenuating after adjusting for socioeconomic and health covariates (41–43). Woolf et al. raised the concern that covariates such as income and health risk factors may themselves be correlated with the predominant policies in Republican-led states, such that adjusting for them in multivariable models could bias estimates toward the null (42). The current study found that the Governor’s political affiliation was consistently associated with all three P-EBT implementation decisions examined, suggesting that the Governor’s affiliation played a role in multiple P-EBT implementation decisions.

This study also contributes to the literature by showing that states that offered more months of SNAP emergency allotments tended to implement P-EBT more consistently in the summer, which is a period of increased food insecurity among children (20, 21, 44–49), and disbursed more P-EBT benefits to eligible households with children. There are several potential reasons for this finding. First, it is possible that states with a longer duration of SNAP emergency allotments had administrative systems better positioned to support P-EBT benefit delivery. Our finding that states with fewer months of prior-year SNAP emergency allotments were less likely to consistently implement Summer P-EBT is congruent with findings from earlier studies of the Summer Electronic Benefit Transfers for Children (SEBTC) demonstration project, an earlier iteration of the school-based summer food program. As examples, earlier evaluation studies showed that SEBTC benefit use was higher among programs that integrated student eligibility data with administrative records from other safety-net programs, such as SNAP and Medicaid, and among children with prior SNAP participation during the school year (21, 46–48). These findings suggest that administrative data integration and prior participation in safety-net programs may facilitate program enrollment and benefit disbursement (i.e., uptake). Second, decisions to de-implement SNAP emergency allotments and P-EBT earlier than required by federal statute were typically made by the Governor of each state. Therefore, it seems plausible that these decisions were correlated because they shared a common source (42).

Importantly, measures of the need for food assistance were not typically linked to P-EBT implementation practices. The poverty rate was not associated with any outcome in bivariate models, multivariable models, or sensitivity analyses. This is notable because poverty remains a well-established structural correlate of food insecurity (16, 50–53). National estimates indicate that approximately 44% of households with children living in poverty experience food insecurity, a rate more than four times the rate observed among households with incomes above 200% of the FPL (54), demonstrating a disproportionately high need for food assistance among households in poverty. Similarly, the unemployment rate was not associated with P-EBT implementation patterns in multivariable models, further suggesting that indicators of economic need were not consistently reflected in program outcomes.

There are two potential reasons why higher Medicaid participation rates are associated with larger per capita P-EBT disbursements. First, this finding is consistent with evidence that enrollment in one safety-net program, such as Medicaid, may facilitate participation in others, such as P-EBT (55–61). Leschewski et al. (55) found that receiving Medicaid benefits was associated with greater nutrition assistance program participation among eligible households, with proposed mechanisms including categorical eligibility, direct certification, and active outreach to inform households of their eligibility and enrollment processes. Tsai et al. (56) similarly found high cross-program take-up between Medicaid and need-based food assistance programs (SNAP), attributing this pattern in part to Medicaid’s relatively lower administrative burden and enrollment accessibility at relevant points of contact, such as schools. Second, it is possible that underlying partisan dynamics within states resulted in differential Medicaid eligibility criteria or accessible enrollment practices, which may have also resulted in comparatively higher per capita P-EBT benefit disbursements among some states (37, 38). Notably, state decisions to expand Medicaid followed partisan lines, with Republican-led states disproportionately opting out, resulting in lower participation rates independent of underlying need (37–39). Variation in income eligibility thresholds and enrollment practices across states further indicates that Medicaid participation rates partly reflect policy generosity rather than poverty alone. Regardless of the underlying mechanism, whether need, policy context, or both, findings from the current study alongside those from prior studies suggest that Medicaid participation reflects a combination of concentrated low-income populations and more expansive state policy environments, both of which may have increased the likelihood that eligible children successfully enrolled in P-EBT (55, 56).

This study also found that larger states disbursed more benefits through P-EBT in 2020, suggesting a more efficient program launch. Such variation is itself partly structured by state size and administrative capacity, as larger states tend to have more personnel, resources, and institutional infrastructure to rapidly implement new programs. Population size may also correlate with political geography, as larger and more densely populated states tend to have Democratic leadership (60–63). Montez et al. (60) documented that states tend to bundle policies that lean politically left or right, with more generous policy environments associated with better population health outcomes, while Fenelon et al. (61) found that welfare generosity varies substantially across states and tends to be greater in more politically liberal states. Taken together, these studies suggest that population size in our models may be operating partly as a marker of broader state-level administrative and political context (60, 61). These findings suggest that realized per capita P-EBT disbursements may reflect both state-level political context and proxies of need for food assistance. The translation of eligibility into actual benefit receipt appears to have been patterned by pre-existing variation in safety-net infrastructure and administrative capacity rather than being uniform across states (55, 56, 60, 61).

## Policy implications

This study used data from all 50 states and found that, over the duration of the P-EBT program, implementation varied systematically by political context, with Republican gubernatorial leadership associated with later initial disbursements, less consistent summer participation, and lower annual benefit amounts disbursed. We also found that indicators of actual need and economic resources, such as poverty and unemployment rates, showed little association with implementation decisions. These patterns now appear to be repeating with SUN Bucks: as of May 2026, all 12 non-participating states are led by Republican Governors, despite robust and accumulating evidence of the program’s impact (4, 18, 21, 22, 46, 64). P-EBT reduced children’s food hardship by 8 percentage points within 2 weeks of disbursement in 2020, lifting an estimated 2.7–3.9 million children out of hunger (4). Research on SNAP, with a structurally identical delivery mechanism to P-EBT, shows reductions in child food insecurity, improved academic outcomes, and long-term gains in health and earnings (65, 66). Each dollar in benefits generates approximately $1.54 in local economic activity (67, 68). These findings underscore that decisions about whether to participate in SUN Bucks are not politically neutral: they may have material consequences for the families with the least capacity to absorb food insecurity.

## Conclusion

Across all 50 states and the District of Columbia, and throughout the P-EBT implementation period, P-EBT implementation varied according to political context and economic conditions and showed little evidence of alignment with indicators of the need for food assistance. These findings have implications for the successful implementation of future food assistance programs and may be translated to the SUN Bucks program, which is implemented using many of the same practices as P-EBT.

## Data Availability

The raw data supporting the conclusions of this article will be made available by the authors, without undue reservation.

## References

[1] Office of the Federal Register. Public Law 116-127 - Families First Coronavirus Response Act (2014).

[2] U.S. Department of Agriculture. SUN Bucks (Summer EBT) (2026). Available online at: https://www.fns.usda.gov/summer/sunbucks (Accessed February 26, 2026).

[3] Bauer L Ruffini K Schanzenbach DW. The effects of lump-sum food benefits during the COVID-19 pandemic on spending, hardship, and health. J Public Econ. (2024) 240:105269. doi: 10.1016/j.jpubeco.2024.105269, 38826717

[4] Bauer L Pitts A Ruffini K Schanzenbach DW (2020). The Effect of Pandemic EBT on Measures of Food Hardship. Available online at: https://www.brookings.edu/wp-content/uploads/2020/07/P-EBT_LO_7.30.pdf (Accessed September 2, 2025)

[5] USDA (2025). State Guidance on Pandemic EBT. Available online at: https://www.fns.usda.gov/snap/state-guidance-coronavirus-pandemic-ebt-pebt (Accessed September 2, 2025)

[6] Jones JW (2021). COVID-19 Working Paper: Supplemental Nutrition Assistance Program and Pandemic Electronic Benefit Transfer Redemptions During the Coronavirus Pandemic.

[7] Jones JW Toossi S (2024). The Food and Nutrition Assistance Landscape: Fiscal Year 2023 Annual Report. Available online at: https://www.ers.usda.gov/publications/pub-details?pubid=109313 (Accessed May 2, 2026)

[8] Kimbro RT Denney JT. Transitions into food insecurity associated with behavioral problems and worse overall health among children. Health Aff. (2015) 34:1949–55. doi: 10.1377/hlthaff.2015.0626, 26526254

[9] Thomas MMC Miller DP Morrissey TW. Food insecurity and child health. Pediatrics. (2019) 144:4. doi: 10.1542/peds.2019-0397, 31501236

[10] Knowles M Rabinowich J Ettinger de Cuba S Cutts DB Chilton M. “Do you Wanna breathe or eat?”: parent perspectives on child health consequences of food insecurity, trade-offs, and toxic stress. Matern Child Health J. (2016) 20:25–32. doi: 10.1007/s10995-015-1797-8, 26156827 PMC4712223

[11] Kinsey EW Oberle M Dupuis R Cannuscio CC Hillier A. Food and financial coping strategies during the monthly supplemental nutrition assistance program cycle. SSM Popul Health. (2019) 7:100393. doi: 10.1016/j.ssmph.2019.100393, 31016223 PMC6468142

[12] Tamiru D Belachew T. The association of food insecurity and school absenteeism: systematic review. Agric Food Secur BioMed Central Ltd. (2017) 6:5. doi: 10.1186/s40066-016-0083-3, 38164791

[13] Coughenour C Conway Kleven B Gakh M Stephen H Chien LC Labus B . School absenteeism is linked to household food insecurity in school catchment areas in southern Nevada. Public Health Nutr. (2021) 24:5074–80. doi: 10.1017/S136898002100063X, 33583473 PMC10200410

[14] McLaughlin KA Greif Green J Alegría M . Food insecurity and mental disorders in a national sample of U.S. adolescents. J Am Acad Child Adolesc Psychiatry. (2012) 51:1293–303. doi: 10.1016/j.jaac.2012.09.009, 23200286 PMC3632292

[15] Baiden P Labrenz CA Thrasher S Asiedua-Baiden G Harerimana B. Adverse childhood experiences and household food insecurity among children aged 0-5 years in the USA. Public Health Nutr. (2021) 24:2123–31. doi: 10.1017/S1368980020002761, 32867879 PMC10195571

[16] Hartline-Grafton H Hassink S. Food insecurity and health: practices and policies to address food insecurity among children. Acad Pediatr. (2021) 1:139–40. doi: 10.1016/j.jses.2017.08.002, 32653691 PMC7347342

[17] Koné Consulting (2020). Pandemic EBT Implementation Documentation Project. Available online at: https://www.cbpp.org/sites/default/files/atoms/files/10-7-20fa-kone.pdf (Accessed September 3, 2025)

[18] Gupta P Gutierrez E Meltzer A Tezel B (2025). Implementation Insights and Equity for Summer EBT in 2024. Available online at: https://www.urban.org/research/publication/implementation-insights-and-equity-considerations-summer-ebt-2024 (Accessed September 2, 2025)

[19] Steffen DR Kim DD. The effects of SNAP emergency allotments on state-level SNAP benefits and enrollment during the COVID-19 pandemic. Health Affairs Scholar. 2:qxae109 doi: 10.1093/haschl/qxae109, 39328393 PMC11426163

[20] Huang J Barnidge E. Low-income children’s participation in the National School Lunch Program and household food insufficiency. Soc Sci Med. (2016) 150:8–14. doi: 10.1016/j.socscimed.2015.12.020, 26722983

[21] Klerman JA Wolf A Collins A Bell S Briefel R. The effects the summer electronic benefits transfer for children demonstration has on children’s food security. Appl Econ Perspect Policy. (2017) 39:516–32. doi: 10.1093/aepp/ppw030

[22] USDA Food Nutrition Service Sun Bucks (Summer EBT). Available online at: https://www.fns.usda.gov/summer/sunbucks (Accessed February 25, 2026)

[23] Grogan CM RE. Federalism partisan politics, and shifting support for state flexibility: the case of the U.S. state children’s health insurance program. Publius: J. Federalism. (2008) 39:47–69. doi: 10.1093/publius/pjn031

[24] John P. The Oxford Handbook of Classics in Public Policy and Administration. Oxford: Oxford University Press (2016).

[25] Rigby E Haselswerdt J. Hybrid federalism, partisan politics, and early implementation of state health insurance exchanges. Publius. (2013) 43:368–91. doi: 10.1093/publius/pjt012

[26] Pollack Porter KM Rutkow L McGinty EE. The importance of policy change for addressing public health problems. Public Health Rep. (2018) 133:9S–14S. doi: 10.1177/0033354918788880, 30426876 PMC6243447

[27] Mena-Carrasco F Barolín N Pollard C Batchelder A Guilamo-Ramos V Szanton S. Building nurse leaders: integrating policy into nursing education. Policy Polit Nurs Pract. (2025) 26:280–9. doi: 10.1177/15271544251346136, 40518966

[28] Census Bureau U.S. (2026). Poverty thresholds. Available online at: https://www.census.gov/data/tables/time-series/demo/income-poverty/historical-poverty-thresholds.html. (Accessed February 19, 2026).

[29] USDA. SNAP Tables. Available online at: https://www.fns.usda.gov/pd/supplemental-nutrition-assistance-program-snap (Accessed May 14, 2026).

[30] USDA. Child Nutrition Programs Income Eligibility Guidelines. Available online at: https://www.fns.usda.gov/schoolmeals/income-eligibility-guidelines (Accessed April 9, 2026).

[31] USAFacts. What is the federal poverty level? Available online at: https://usafacts.org/explainers/what-is-the-federal-poverty-level/country/united-states/ (Accessed February 6, 2026).

[32] USDA. Community Eligibility Provision. Available online at: https://www.fns.usda.gov/cn/cep (Accessed February 19, 2026).

[33] U.S. Bureau of Economic Analysis (2026). Technical Notes on Regional Price Parities and Implicit Regional Price Deflators. Available online at: https://www.bea.gov/news/technical-notes-regional-price-parities-and-implicit-regional-price-deflators (Accessed June 28, 2026)

[34] U.S. Bureau of Economic Analysis (2026). 2019 Regional Price Parities by State. Available online at: https://apps.bea.gov/itable/?ReqID=99&step=1#eyJhcHBpZCI6OTksInN0ZXBzIjpbMSwyOSwyNSwyNiwyNyw0MF0sImRhdGEiOltbIlRhYmxlSWQiLCIxMDEiXSxbIk1ham9yQXJlYUtleSIsIjAiXSxbIkxpbmUiLCIxIl0sWyJTdGF0ZSIsIjAiXSxbIlVuaXRfb2ZfTWVhc3VyZSIsIkxldmVscyJdLFsiTWFwQ29sb3IiLCJCRUFTdGFuZGFyZCJdLFsiblJhbmdlIiwiNSJdLFsiWWVhciIsIjIwMjMiXSxbIlllYXJCZWdpbiIsIi0xIl0sWyJZZWFyRW5kIiwiLTEiXV19 (Accessed June 28, 2026)

[35] Aten BH. Regional Price parities and real regional income for the United States. Soc Indic Res. (2017) 131:123–43. doi: 10.1007/s11205-015-1216-y

[36] Gascon CS (2014). Overview: Buying Power of Minimum Wage Varies Across and Within States. Available online at: https://www.stlouisfed.org/publications/regional-economist/october-2014/buying-power-of-minimum-wage-varies-across-and-within-states (Accessed June 30, 2026)

[37] Grant R. The triumph of politics over public health: states opting out of medicaid expansion. Am J Public Health. (2014) 104:203–5. doi: 10.2105/AJPH.2013.301717, 24328625 PMC3935692

[38] Grogan CM. Medicaid’s political development since 1965: how a fragmented and unequal program has expanded. J Health Polit Policy Law. (2025) 50:137–64. doi: 10.1215/03616878-11567692, 39327778

[39] KFF (2026). Status of State Medicaid Expansion Decisions. Available online at: https://www.kff.org/medicaid/status-of-state-medicaid-expansion-decisions/ (Accessed May 3, 2026)

[40] University of Kentucky Center for Poverty Research (1980-2024). UKCPR National Welfare Data. Available online at: https://ukcpr.uky.edu/resources#National-Welfare-Data (Accessed February 25, 2026)

[41] Shvetsova O Zhirnov A Giannelli FR Catalano MA Catalano O. Governor’s party, policies, and COVID-19 outcomes: further evidence of an effect. Am J Prev Med. (2022) 62:433–7. doi: 10.1016/j.amepre.2021.09.003, 34756754 PMC8502787

[42] Woolf SH Sabo RT Chapman DA Lee JH. Association between partisan affiliation of state governments and state mortality rates before and during the COVID-19 pandemic. Milbank Q. (2023) 101:1191–222. doi: 10.1111/1468-0009.12672, 37706227 PMC10726914

[43] Neelon B Mutiso F Mueller NT Pearce JL Benjamin-Neelon SE (2021). Associations between governor political affiliation and COVID-19 cases, deaths, and testing in the United States. Am J Prev Med. 61:115–119. doi:10.1016/j.amepre.2021.01.034, 33775513 PMC8217134

[44] Fern SE Kimbro RT Hill ME Hughes CC. Emergency food support preference and usage during COVID-19: a neighborhood study of low-income black mothers’ use of school-based food distribution and P-EBT. Am J Public Health. (2023) 113:S227–30. doi: 10.2105/AJPH.2023.307458, 38118086 PMC10733869

[45] Acciai F Ohri-Vachaspati P Yedidia MJ. Schools’ participation in the community eligibility provision affects students’ receipt of emergency benefits during the COVID-19 pandemic. Nutrients. (2022) 14:4919. doi: 10.3390/nu14224919, 36432605 PMC9693532

[46] Gordon AR Briefel RR Collins AM Rowe GM Klerman JA. Delivering summer electronic benefit transfers for children through the supplemental nutrition assistance program or the special supplemental nutrition program for women, infants, and children: benefit use and impacts on food security and foods consumed. J Acad Nutr Diet. (2017) 117:367–375.e2. doi: 10.1016/j.jand.2016.11.002, 28017594

[47] Nutter R Enver A Porowski A Wei W Weiss C Chanhatasilpa C . Evaluation of the 2019–2022 Summer EBT Demonstration. Alexandria, VA: U.S. Department of Agriculture, Food and Nutrition Service, Office of Policy Support (2024). Available online at: https://fns-prod.azureedge.us/sites/default/files/resource-files/ops-sebt-2019-2022-report.pdf (Accessed February 25, 2026)

[48] Nutter R Enver A Boyle M Thompson M Williams K Chanhatasilpa C . Summer Electronic Benefit Transfer for Children Evaluation, 2015–18. Alexandria, VA: U.S. Department of Agriculture, Food and Nutrition Service, Office of Policy Support (2024). Available online at: https://fns-prod.azureedge.us/sites/default/files/resource-files/ops-sebt-2015-2018-report.pdf (Accessed February 25, 2026)

[49] Chen Y Hager ER Gross J Gross SM. The interplay between summer meals, food insecurity, and diet quality among low-income children in Maryland, USA: a multiphase cross-sectional study. Nutrients. (2025) 17:2055. doi: 10.3390/nu17132055, 40647162 PMC12251470

[50] Pinard CA Bertmann FMW Byker Shanks C Schober DJ Smith TM Carpenter LC . What factors influence SNAP participation? Literature reflecting enrollment in food assistance programs from a social and behavioral science perspective. J Hunger Environ Nutr. (2017) 12:151–68. doi: 10.1080/19320248.2016.1146194

[51] Higashi RT Sood A Conrado AB Shahan KL Leonard T Pruitt SL. Experiences of increased food insecurity, economic and psychological distress during the COVID-19 pandemic among supplemental nutrition assistance program-enrolled food pantry clients. Public Health Nutr. (2022) 25:1027–37. doi: 10.1017/S1368980021004717, 34865672 PMC8712963

[52] Berkowitz SA Seligman HK Palakshappa D. Understanding food insecurity risk in the United States: a longitudinal analysis. SSM Popul Health. (2024) 25:101569. doi: 10.1016/j.ssmph.2023.101569, 38156292 PMC10753081

[53] Keith-Jennings B Llobrera J Dean S. Links of the supplemental nutrition assistance program with food insecurity, poverty, and health: evidence and potential. Am J Public Health. (2019) 109:1636–40. doi: 10.2105/AJPH.2019.305325, 31693420 PMC6836787

[54] Rabbitt MP Reed-Jones M Hales LJ Suttles S Burke MP (2025). Household Food Security in the United States in 2024. Available online at: www.ers.usda.gov (Accessed February 25, 2026)

[55] Leschewski A Davis DE. Nutrition assistance program take-up given multiprogram eligibility. J Agric Appl Econ Assoc. (2023) 2:35–50. doi: 10.1002/jaa2.44

[56] Tsai MM Yeb JA Jackson KE Gosliner W Fernald LCH Hamad R. Understanding multiprogram take-up of safety net programs among California families. AJPM Focus. (2024) 3:100216. doi: 10.1016/j.focus.2024.100216, 38638939 PMC11024909

[57] Tsai MM Fernald LCH Hamad R Jackson KE Fernández-Viña N Bradshaw PT . Safety net program participation patterns, sociodemographic factors, and health. Am J Prev Med. (2025) 68:964–73. doi: 10.1016/j.amepre.2025.01.027, 39914646 PMC12033079

[58] Newman C Todd J Ver PM. Children’s participation in multiple food assistance programs: changes from 1990 to 2009. Soc Serv Rev. (2011) 85:535–64. doi: 10.1086/663833

[59] Hardy B Hill HD Romich J. Strengthening social programs to promote economic stability during childhood. Child Policy Nexus. (2019) 32:1–36. doi: 10.1002/sop2.4, 32523328 PMC7286602

[60] Montez JK Grumbach JM. US state policy contexts and population health. Milbank Q. (2023) 101:196–223. doi: 10.1111/1468-0009.12617, 37096608 PMC10126966

[61] Fenelon A Witko C. Emerging political and demographic divides: state politics, welfare generosity, and adult mortality in U.S. states 1977–2017. Health Place. (2021) 71:102644. doi: 10.1016/j.healthplace.2021.102644, 34352496 PMC8490313

[62] Eubank N Rodden J. Who is my neighbor? The spatial efficiency of partisanship. Stat Public Policy. (2020) 7:87–100. doi: 10.1080/2330443X.2020.1806762

[63] Wilkinson W. The Density Divide: Urbanization, Polarization, and Populist Backlash (2019). Available online at: www.niskanencenter.org (Accessed February 25, 2026)

[64] Bleich SN Orona KC Turner L. New federal program to address child hunger begins this summer. JAMA Health Forum. (2024) 5:E242133. doi: 10.1001/jamahealthforum.2024.2133, 38842798

[65] Leung CW Wolfson JA. The impact of the thrifty food plan benefit re-evaluation on SNAP participants’ short-term food security and health outcomes. Front Public Health. (2021) 11:11. doi: 10.3389/fpubh.2023.1142577, 37457281 PMC10343438

[66] Gundersen C Kreider B Pepper JV. Partial identification methods for evaluating food assistance programs: a case study of the causal impact of snap on food insecurity. Am J Agric Econ. (2017) 99:875–93. doi: 10.1093/ajae/aax026

[67] Canning P Mentzer Morrison R. Quantifying the impact of SNAP benefits on the U.S. Econ Jobs (2019). Available online at: https://www.ers.usda.gov/amber-waves/2019/july/quantifying-the-impact-of-snap-benefits-on-the-u-s-economy-and-jobs (Accessed May 2, 2026)

[68] Hanson K. (2010). The Food Assistance National Input-Output Multiplier (FANIOM) Model and the Stimulus Effects of SNAP. Available online at: https://www.ers.usda.gov/publications/pub-details?pubid=44749

