# Supplementary material for: National associations between pandemic electronic benefit transfer (P-EBT) implementation practices and state characteristics
Source: Front Public Health. 2026 Aug 10;14:1886776. doi: 10.3389/fpubh.2026.1886776 (PMC13500631; doi:10.3389/fpubh.2026.1886776)
Supplement: Supplementary file 1 [file Table_1.docx]

**Supplemental Table 1**

State characteristics by Governor's political party affiliation, 2020-2023.

| **Characteristic** | **Democratic Governor**  **Mean (SD)** | **Republican Governor**  **Mean (SD)** | **Difference**  **(Dem – Rep)** | **p value** |
| --- | --- | --- | --- | --- |
| Medicaid Participation Rate | 27.6% (7.3%) | 22.6% (6.4%) | 5% | <0.001 |
| Per Capita GSP^a^ (1=lowest, 4=highest) | 2.85 (1.03) | 2.33 (1.11) | 0.52 | <0.001 |
| Unemployment Rate | 5.86 (2.24%) | 4.55 (1.77%) | 1.31% | <0.001 |
| State Minimum Wage ($) | $10.49 (2.45) | $8.63 (2.10) | $1.86 | <0.001 |
| Poverty Rate^b^ | 10.96% (3.03%) | 11.30% (3.01%) | - 0.34% | <0.05 |
| Population Size | 7,361,715 (8,105,761) | 5,758,763 (6,488,922) | 1,602,952 | <0.001 |
| Months with SNAP Emergency Allotments | 5.52 (3.62) | 4.42 (3.57) | 1.10 | <0.001 |

^a^ Per capita GSP coded as quartiles (1=lowest, 4=highest); values reflect mean quartile rank.

^b^ Republican-led states had higher average rates than Democratic-led states, resulting in a negative difference between Democratic-led vs Republican-led.

Note: Means reflect averages across all state-year observations from 2020-2023 (n=1,887). All independent variables are lagged one year relative to outcomes. Differences tested using two-sample t-tests

**Supplemental Table 2**

Multivariable Associations Between State Characteristics and P-EBT Program Implementation Outcomes, Using Real Minimum Wage

|  | **Month of First P-EBT Disbursement in 2020^a^  (n=51)** | | **Inconsistent Summer P-EBT Participation 2022- 2023^b^  (n=51)** | | **Annual per-Capita P-EBT Disbursements ($) Dec. 2020-2023 (n=204)** | |
| --- | --- | --- | --- | --- | --- | --- |
| **Variable (t-1)** | **Odds Ratio (OR)** | **95% Confidence Interval** | **Odds Ratio (OR)** | **95% Confidence Interval** | **Coefficient (SE)^c^** | **p value for Year ×  t-1 Variable Interactions^d^** |
| Medicaid Participation Rate | 0.0013 | (<0.001, 385.7) | 0.01 | ---^f^ | 2290.2 (1446.0) | 0.97 |
| Per Capita GSP ($) |  |  |  |  |  | 0.34 |
| Quartile 1   (ref.) | --- | --- | --- | --- | --- | --- |
| Quartile 2 | 1.59 | (0.34, 7.29) | 2.36 | (0.10, 54.32) | 163.1 (215.5) | --- |
| Quartile 3 | 3.36 | (0.58, 19.35) | 1.29 | (0.01, 140.5) | - 197.3 (160.1) | --- |
| Quartile 4 (highest) | 1.83 | (0.188, 17.96) | 14.8 | (0.05, 4260.3) | 435.5 (294.3) | --- |
| Unemployment Rate | 1.01 | (0.67, 1.50) | 0.93 | (0.19, 4.45) | 47.4 (40.9) | 0.84 |
| Real Minimum Wage | 0.93 | (0.63, 1.37) | 0.91 | (0.53, 1.56) | - 32.8 (44.8) | **0.041** |
| Poverty Rate | 1.21 | (0.93, 1.58) | 1.25 | (0.72, 2.16) | - 56.5 (31.6) | **0.031** |
| Population Size | 0.99 | (0.99, 1.00) | 1.00 | (0.99, 1.00) | **0.0000398 (0.000011)**** | **<0.0001** |
| Months with SNAP Emergency Allotments^e^ | --- | --- | **0.71** | **(0.56, 0.90)**** | - 26.9 (61.3) | 0.20 |
| Republican Governor^b^ | 2.07 | (0.57, 7.49) | --- | --- | - 42.4 (181.9) | 0.64 |

Footnotes: All independent variables were lagged one year relative to the outcome. However, models for inconsistent summer participation used 2021 covariate values to ensure exposure preceded the first potential occurrence of inconsistent participation in 2022. Real Minimum Wage is the state minimum wage (t-1) adjusted by the 2019 U.S. BEA Regional Price Parity (RPP) for that state (real_minwage = lagged minimum wage / (RPP/100)).

* p < 0.05, ** p < 0.005

^a^ Estimates were obtained from an ordered logistic regression model and month of first disbursement was captured as a calendar month, ranging from March (3) to August (8). So, an odds ratio <1 indicates earlier payment timing and odds ratios >1 indicate later payment timing.

^b^ Estimates were obtained from a logistic regression model. Models for inconsistent Summer P-EBT participation were estimated using independent variable values from 2021, thus ensuring these occurred prior to the outcome assessment. Republican Governor variable was excluded due to perfect prediction of the outcome; only states with Republican Governors inconsistently participated in Summer P-EBT.

^c^ Estimates for annual per capita disbursements were obtained from pooled linear regression models using December observations for all U.S. states and Washington, D.C., from 2020 through 2023. Year indicator variables were included to model time. Standard errors were clustered at the state level.

^d^ Estimates for annual per capita disbursements were obtained from pooled linear regression models using December observations for all U.S. states and Washington, D.C., from 2020 through 2023. Year indicator variables were included to model time. Model included interactions between year and all covariates, and joint Wald tests assessed whether associations differed across years. Standard errors were clustered at the state level.

^e^ SNAP Emergency Allotments were implemented in 2020, and all states implemented the first year of issuance. Therefore, this variable was not included in the first month disbursement model.

^f^ Confidence interval unreliable due to near-complete separation.

**Supplemental Table 3**

Multivariable Associations Between State Characteristics and per-capita P-EBT Disbursements Using Real Minimum Wage, Stratified by Year (2020-2023) (n=51)

| **Variable (t-1)** | **2020**  **Coef. (SE)** | **2021**  **Coef. (SE)** | **2022**  **Coef. (SE)** | **2023**  **Coef. (SE)** |
| --- | --- | --- | --- | --- |
| R² | **0.53***** | **0.58***** | 0.32 | 0.29 |
| Medicaid Participation Rate | 2300.86 (1526.7) | 2130.30 (2283.1) | **1816.70 (872.8)*** | **2276.27 (841.6)*** |
| Per Capita GSP ($) |  |  |  |  |
| Quartile 1 (ref.) | --- | --- | --- | --- |
| Quartile 2 | 168.75 (182.5) | 160.60 (261.1) | 65.42 (134.8) | 220.61 (186.1) |
| Quartile 3 | - 191.49 (200.7) | 367.59 (328.0) | 216.83 (151.6) | 294.74 (187.2) |
| Quartile 4 (highest) | 432.82 (270.4) | 61.50 (311.7) | 263.41 (160.4) | 375.24 (195.4) |
| Unemployment Rate | 46.04 (43.0) | 18.92 (115.2) | - 23.92 (76.7) | 53.92 (69.3) |
| Real Minimum Wage | - 32.90 (46.7) | **163.45 (53.1)**** | - 1.44 (21.1) | - 8.72 (18.7) |
| Poverty Rate | - 57.69 (32.1) | 9.86 (41.5) | 21.30 (19.7) | - 31.48 (19.6) |
| Population Size | **0.0000397**  **(0.00000928)***** | 0.0000139  (0.0000120) | - 0.00000048  (0.00000549) | - 0.0000038  (0.00000569) |
| Months with SNAP Emergency Allotments^a^ | --- | **112.48 (52.6)*** | 16.14 (10.1) | 25.30 (47.8) |
| Republican Governor^b^ | - 34.33 (155.1) | - 174.95 (198.4) | 120.10 (98.6) | 185.67 (106.3) |

Footnotes: All independent variables were lagged one year relative to the outcome. Real Minimum Wage is the state minimum wage (t-1) adjusted by the 2019 U.S. BEA Regional Price Parity (RPP) for that state (real_minwage = lagged minimum wage / (RPP/100)).

* p < 0.05, ** p < 0.005 *** p < 0.0005

^a^ SNAP Emergency Allotments were implemented in 2020, and all states implemented the first year of issuance. Therefore, this variable was not included in the 2020 model.

^b^ Linear regression models were estimated separately for each year using December state level observations (n = 51 per year). Standard errors were clustered at the state level.
